# Coating of Mesh Grafts for Prolapse and Urinary Incontinence Repair with Autologous Plasma: Exploration Stage of a Surgical Innovation

**DOI:** 10.1155/2014/296498

**Published:** 2014-09-16

**Authors:** Dimitri Barski, Holger Gerullis, Evangelos Georgas, Andreas Bär, Bernhard Lammers, Albert Ramon, Dirk Ysebaert, Bernd Klosterhalfen, Mihaly Boros, Thomas Otto

**Affiliations:** ^1^Department of Urology, Lukas Hospital, 41464 Neuss, Germany; ^2^German Centre for Assessment and Evaluation of Innovative Techniques in Medicine (DZITM), 41464 Neuss, Germany; ^3^Department of Surgery, Lukas Hospital, 41464 Neuss, Germany; ^4^ITERA (International Tissue Engineering Research Association), 2000 Antwerpen, Belgium; ^5^Department of Surgery, University of Antwerpen, 2000 Antwerpen, Belgium; ^6^German Centre for Implant-Pathology, 52351 Düren, Germany; ^7^Department of Experimental Surgery, University of Szeged, H-6720 Szeged, Hungary

## Abstract

*Purpose*. Optimized biocompatibility is a major requirement for alloplastic materials currently applied for stress urinary incontinence (SUI) and pelvic organ prolapse (POP) repair. In the preliminary studies the mesh modification by coating with autologous plasma resulted in the increased adherence score *in vitro* and improved biocompatibility in an animal model. The first use of plasma coated meshes in human is presented. *Materials and Methods*. Between 04/2013 and 05/2014, 20 patients with the indication for SUI and POP repair were selected in a single institution. The applied meshes were modified by autologous plasma coating prior to implantation. A retrospective chart review for peri- and early postoperative complications was performed. Functional outcome and QoL were evaluated pre- and postoperatively. *Results*. The functional outcome and QoL improved significantly in all groups. Two reoperations (Grade IIIB) with the release of TVT-mesh in anesthesia due to the obstruction were needed. No other severe complications were registered. *Conclusion*. For the first time we applied a mesh modification in a human setting according to *IDEAL* criteria of surgical innovations. The procedure of mesh coating with autologous plasma is safe and a prospective randomized trial proving a positive effect of plasma coating on the biocompatibility and morbidity outcome with long-term registry is planned.

## 1. Introduction

Currently the approval of medical devices as surgical meshes is regulated by American Food and Drug Administration (FDA) and European guidelines according to risk classification. Clinical trials and postmarket followup were not required for the commercial approval. In a Public Health Notification (PHN), from 2008, the FDA reported more than 1000 unexpected and severe adverse events, associated with transvaginal placement of surgical mesh to treat POP and SUI. In 2011, a second FDA warning has been amended, proposing an upgrading in risk classifications for meshes, which would allow the request of premarket approval and postmarket surveillance studies [1].

Meshes or grafts potentially add to the complication profile the aspects of trauma of insertion, foreign body reaction to the implant in terms of inflammation, infection and/or rejection, and the stability of the prosthesis over time [2]. Polypropylene meshes (Type 1, Amid-classification) are usually used for vaginal repair of POP and SUI [3]. The rate of mesh-related complications after transvaginal mesh application for POP is about 15–25% and especially mesh erosion up to 10% for these indications [4, 5]. Most common complications after MUS (midurethral sling) are obstruction,* de novo* urge, chronic pain, dyspareunia, and mesh erosion [6]. The complications are attributed to a considerable extent to the wrong indication and faulty surgical techniques; material properties are the other reasons. The choice of the optimal mesh for a particular indication with the highest functionality (hold shape) as well as minimized side effects remains difficult. Mesh material (type of polymer, pore size, and material weight, etc.) and its biocompatibility were detected to be crucial parameters [7, 8]. A biocompatibility is described by the foreign body reaction (FBR) at the host-tissue/biomaterial interface. The dynamic of the FBR is given by the inflammatory host response depending on the biomaterial composition (Table 1) [7, 9, 10]. The current understanding about an optimized surgical mesh describes a material that permits the transmigration and localisation of beneficial host cells and if directly exposed to visceral organs, vessels, or nerves it strongly inhibits the adherence of the respective organs in order to avoid erosion, foreign body provoked pain, and so forth. Inert (Titan), (partly) absorbable, light-weight materials are currently under development. Sophisticated methods, like preoperative coating of meshes with a protective layer on the visceral side of the mesh, have been frequently investigated, mostly* in vivo* [11, 12]. They seem to present a potential approach to reduce foreign body reaction and improve biocompatibility and therefore have been introduced in mesh applying surgery.

In a considerably narrow time frame, reacting to the first and second FDA warnings, our international scientific collaboration group has recently developed and concluded preliminary studies in order to investigate and improve biocompatibility of surgical meshes. Our entire innovative approach has been conducted following the five-step* IDEAL* model for surgical innovations (*Innovation, Development, Exploration, Assessment, and Long-term study*) with the aim of maintaining it comparable and reproducible at every single step of development [13]. A validated* in vitro* test system to compare biocompatibility features of different meshes has been developed (*Idea*, first stage) [9]. This test system was subsequently expanded, to show that mesh modification by autologous plasma coating results in higher biocompatibility and adherence score* in vitro* [9, 10]. The predictability of these approaches, biocompatibility evaluation, and improvement by plasma coating could then be validated and confirmed in a two-year large animal study (*Development*, second stage) [14]. In particular, an early inflammation reaction seems to be influenced by the coating procedure [15]. Herewith we present a consecutive study on the first clinical assessment of meshes modified by autologous plasma coating in human (*Exploration*, third stage).

## 2. Materials and Methods

**Figure 1:**
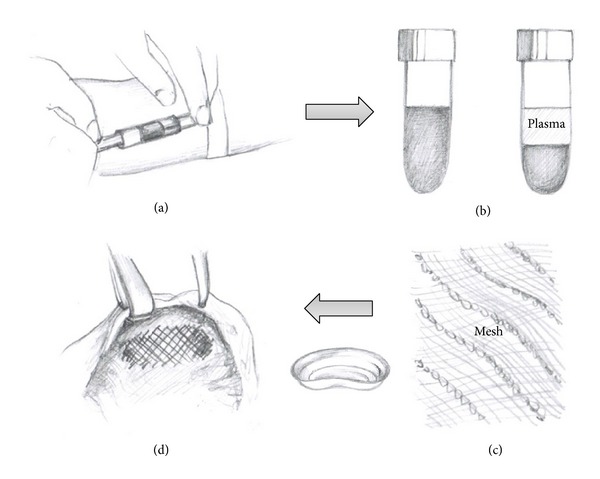
The technique of mesh coating with autologous plasma. (a) Vein puncture, 20–40 mL blood is obtained in EDTA-tube before anesthesia. (b) Centrifugation of blood sample in the operation room. (c) Plasma is abstracted and incubated with the mesh in a bowl. (d) The coated mesh is implanted. The rest of plasma is spilled over the implantation site.

**Figure 2:**
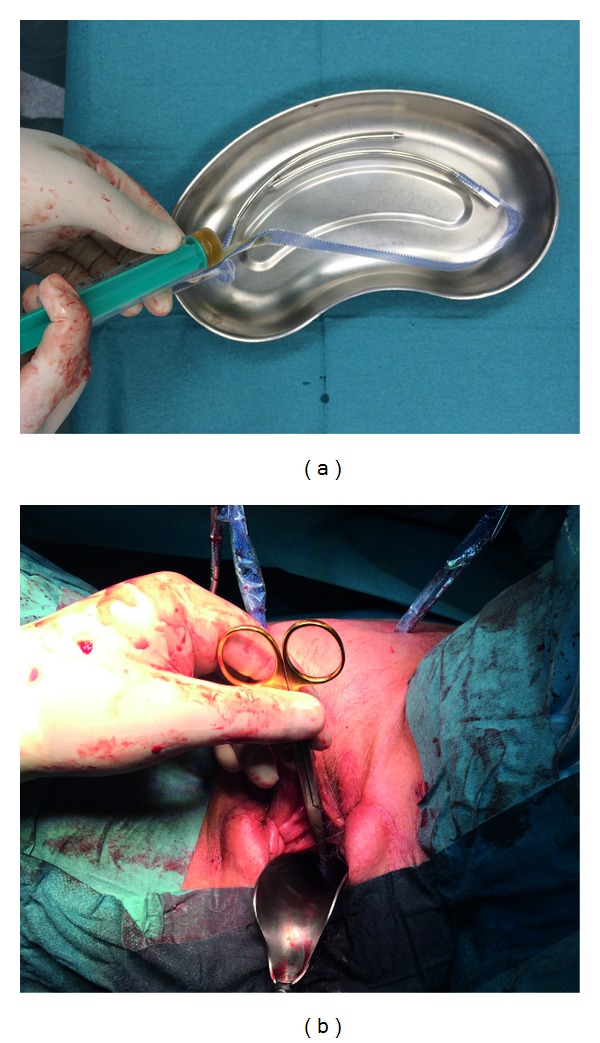
TVT-procedure. (a) Coating of TVT-mesh with autologous plasma. (b) Insertion of retropubic midurethral sling.

Patients (age > 18 y) with surgical indication for SUI (Stamey grade ≥ I) and POP (POP-Q Grades I–III and anterior and apical prolapse) repair with mesh were selected after the informed consent. In case of POP and SUI a concomitant* Burch* colposuspension was performed. All patients experienced an unsuccessful treatment with medicaments and physiotherapy prior to operation. The male patients presented a moderate SUI (grade I-II, 2–6 pads/day) after radical prostatectomy. Urodynamics and urethrocystoscopy were performed prior to the operation and a partial defect of the external sphincter was revealed. According to the* IDEAL* model a sophisticated, well-defined selection of patients was performed. The exclusion criteria were previous mesh implantation at the operation site, infection, chemo- or immunological therapy during the last three months, psychiatric illness or inability to answer the questionnaire, and pregnancy. Different mesh materials were used (TVT, Seratim, Ultrapro, and Vitamesh) (Table 1). 20–40 mL blood sample was obtained in the EDTA-tube (ethylenediaminetetraacetic acid) from the respective patient by vein puncture before the induction of anesthesia. The blood collection and centrifugation of blood sample (4000 rpm for 10 min) was performed in the operation room in order to prevent the contamination. The clear supernatant (plasma) after centrifugation of the precipitation was removed with sterile syringe. Before the implantation the meshes were incubated for 30 min with 10–20 mL (depending on the size of the mesh) autologous plasma in a bowl (Figures 1 and 2). The surgical technique was not altered by the application of this technology (Figure 2). The patients were examined pre- and postoperatively and interviewed before the operation and on telephone 6–8 weeks after the operation. For high grade POP (grade ≥ III) a perioperative ureteral stenting for about two weeks was performed. Ultrasound controls for residual urine volume and hydronephrosis were done after catheter removal on the third postoperative day. In cases of obstruction due to MUS (midurethral sling) a prolonged catheterisation was needed. If the voiding dysfunction persisted (residual volume > 200 mL) an endoscopic evaluation with cystoscopic release of the sling was performed. The patient charts were searched for perioperative and early postoperative complications. The safety of our technology for the patient was validated by the Clavien-Dindo classification of surgical complications and ICS/IUGA classification [2, 16]. The quality of life (QoL) was assessed by P-QOL and ICIQ-SF 2004 questionnaires [17]. In cases of explantation the immunhistochemistry analyses of the mesh are planned [7, 14].

## 3. Legal Requirements

The application of autologous blood plasma coating was performed according to the German Pharmaceutical Law (AMG), the Medical Product Act (MPG) and the Transfusion Act. The permission for this new experimental method was provided by local government. According to the statement of the local government, the preparation of autologous blood plasma and the modification of the mesh by the coating procedure are subject to paragraph 13, 2 b, of the AMG and no permission according to paragraph 13, 1, of the AMG is necessary.

The patients were carefully educated on the experimental technique and possible complications. Because of the retrospective data evaluation no ethical approval was necessary.

## 4. Results

Between 04/2013 and 05/2014, 20 patients (16 females and 4 males) with the indication for SUI and POP repair with mesh graft were selected for surgery in a single institution. The patient characteristics are described in Table 2. The mean age was 67 years (45–85) and the mean followup was 3 months [1–7]. 11 patients were treated for SUI (grades II-III, Stamey score) and 9 patients were treated for POP (POP-Q grades I–III, anterior and apical prolapse). In 50% of patients concomitant operations (*Burch* colposuspension, sacrospinous fixation, and rectopexy) were performed. No intraoperative problems or complications (transfusion reaction, etc.) associated with mesh coating with autologous plasma were observed. Two reoperations (10%, Clavien-Dindo Grade IIIB) with the cystoscopic release of TVT-mesh in anesthesia due to the obstruction were needed. No other severe complications (mesh exposure, bladder or bowel injury, and fistula) were registered. Prolonged perineal paraesthesia and hematoma were observed in 2 cases after TOT (50%) (Table 3). An 85-year female with extended usage of analgesics and antidepressant agents presented a prolonged voiding dysfunction after TVT. Prolonged catheterization and the cystoscopic release were not successful. A suprapubic tube was inserted, the antidepressants were reduced, and the medication with Ubretid was started. A 76-year female presented persisting SUI after the anterior POP repair (grade III) with sacrocolpopexy and consecutive TVT (plasma-coated). The postoperative examination revealed a persisting Grade II-cystocele. A reoperation with colporrhaphy and plasma-coated vaginal mesh application is planned. Two of four male patients after TOT procedure complained about persisting SUI (>1 pad/day); in these cases an artificial urinary sphincter was planned. The functional outcome and QoL improved overall in all groups during the followup. No mesh resections or explantations were necessary up-to-date.

## 5. Discussion

The preliminary work on the principles of plasma coating were described in* in vitro* and animal studies previously [9, 10, 14, 15]. Our study illustrates the first clinical usage of the mesh modification by autologous plasma for POP and SUI repair. The observed early perioperative complications correspond to the data of current meta-analyses and studies [4, 5, 18]. Voiding dysfunction, UTI, recurrent SUI, and paraesthesia were described previously and are associated mostly with the surgical technique and not to the mesh modification. The procedure is safe and offers good functional results. The only Grade III (Clavien-Dindo) complication in the TVT-group was the obstruction with the need of reoperation. This complication is due to the operation technique and has no relation to the coating procedure. The technique of plasma coating is an easy-to-do and timely procedure. No additional complications or intraoperative problems due to this technique were observed. The complications were graduated according to Clavien-Dindo and ICS (International Continence Society)/IUGA (International Urogynecologic Association) classification. The ICS/IUGA classification is based on the information on the category, time, and location of complications. We had problems to make a precise classification for some complications due to inconsistent definitions (Table 3). Because of high complexity and low concordance in different trials ICS/IUGA-classification is currently rarely used [4, 19]. However, we consider the classification to be valuable for the report of long-term data in registries.

The current studies show the importance of acute inflammatory and immune responses for the integration of mesh into the surrounding tissue [9, 10, 15]. Foreign body reaction (FBR) often causes a fibrotic rebuilding of implants and the loss of functions (loss of flexibility, etc.). Furthermore, there is a risk of complications, like deformations (capsule fibrosis of breast implants), chronic pain, and dyspareunia, especially in a sensitive genital region. Seconds after the implantation, the biomaterials are covered by protein layer and 4–8 hours later the macrophages appear and in a few days a granuloma with fibrotic tissue appears [20]. Albumin, fibrinogen (Fg), and immune complexes, in particular IgG, can be found on many surfaces after implantation, such as polyethylene terephthalate (PET), expanded polytetrafluoroethylene (ePTFE), polydimethylsiloxane, polyurethane, and polyethylene polymers, which are all important materials in the manufacture of the implant [7]. Fibrin or fibrinogen modulation by the proteins in the inflammatory response after implantation of foreign materials in the body is particularly important. Studies show that plasma-coated surfaces accumulate significantly less inflammatory cells compared to uncoated surfaces [21, 22]. The profound understanding of the FBR plays the crucial role for optimisation of biocompatibility of alloplastic materials in order to reduce the complications.

An ideal graft material is supposed to be chemically inert, nontoxic, nonallergic, noninflammatory, resistant to infection, noncarcinogenic, solid, sterilizable, convenient, and affordable [8]. New developments in material optimization are currently tested. There are only a few groups who have investigated polypropylene mesh modifications by surface coating with collagen, titanium, or absorbable polymers in animal and* in vitro* studies [11, 12, 23, 24]. While some of these studies found higher biocompatibility (e.g., light polypropylene mesh) compared to the standard polypropylene control group, others found very similar outcomes between the two groups. Some of these meshes have been now introduced into the market as they were thought to be associated with lower complications [25]. Our study group was the first one to analyse the mesh modification according to* IDEAL* criteria of surgical innovation [13]. On the basis of the results presented in this study we are currently initiating a prospective randomised clinical trial for the optimization of implants in mesh surgery. We will compare the group of native meshes versus coated meshes for postoperative complications and functional results. The last step of* IDEAL* model with long-term surveillance of mesh grafts was successfully introduced for hernia surgery by national and European registries [26, 27]. A consecutive urogynecological registry for implants is currently under construction (unpublished data).

It is crucial that randomised controlled clinical trials should be supported in the future, in particular with regard to fundraising or industrial sponsoring. Therefore research funders need to recognise the nature of surgical innovation to encourage high-quality research approaches.

In the study presented here we could first transfer the previous* in vitro* and animal model findings on optimisation of mesh properties in human. The results of this research and the developed evaluation approach for meshes could get more important in the future evaluating processes as the method can be performed independent from manufacturers concerns, in particular after market entry [14].

## 6. Conclusion

Coating of meshes with autologous plasma prior to implantation is a safe procedure with no increased perioperative complications. The modification is implemented according to* IDEAL *criteria of surgical innovations (*Exploration* stage). A randomized single-blinded clinical trial proving a positive effect of plasma coating on the biocompatibility of meshes and morbidity outcome is justified and is in the progress of preparation (*Assessment stage*). A long-term surveillance of new mesh materials will be performed in national and European urogynecological registries (unpublished data, EuraHS) (*Long-Term* stage). In reaction to FDA reports on mesh associated problems, our international collaboration group presents a unique implementation of all five steps of surgical innovations for mesh graft development in urogynecology.

## Figures and Tables

**Table 1:** Material and biomechanic characteristics of selected meshes [7, 9, 10, 14, 28–31].

Mesh	Material	Biomechanic characteristics	Adhesion score (Melman)	Adhesion score after coating with plasma (Melman)
Seratim PA, Serag Wiessner	Monofilament polypropylene, polyglycol acid, and caprolacton	Partly absorbable (90–120 days) Pore size: 5800 *µ*m (11 mm^2^) Weight: 15 g/m^2^ (after resorption) Thickness: 0.5 mm Tear resistance (*F*max): 80 N	2.5	Pending

Vitamesh, ProxyBiomedical	Monofilament polypropylene	Nonabsorbable Weight: 35 g/m^2^ Pore size: 2410 *µ*m Thickness: 0.25 mm Tear resistance (*F*max): 33.7 N	1.6	1.9

UltraPro, Ethicon	Monofilament polypropylene reinforced with poliglecaprone fibers (Monocryl)	Partly absorbable (90–120 days) Pore size: 3000–4000 *µ*m Weight: 28 g/m^2^ (after resorption) Thickness 0.5 mm Tear resistance (*F*max): 69 N	1.4	1.6

TVT, Johnson and Johnson	Monofilament polypropylene	Nonabsorbable Pore size: <1000 *µ*m Weight: 105–110 g/m^2^ Thickness: 0.7 mm Tear resistance (*F*max): about 10N	1	1.6

**Table 2:** Patient characteristics.

Procedure	TVT	TOT	Anterior vaginal mesh	Sacrocolpopexy
Number of patients (gender)	7 (female)	4 (male)	1 (female)	8 (female)
Age, mean (yr)	67 (57–85)	71 (70–72)	58	64 (45–75)
Operation time, mean (min)	36 (31–49)	46 (42–55)	51	57 (43–71)
Followup, median (mos)	3 (2–4)	4 (2–7)	3	3 (1–4)
Concomittant procedures	1 × SSF	No	1 × SSF	8 × *Burch*, 1 × Rectopexy

SSF: sacrospinous fixation.

**Table 3:** Peri- and early postoperative complications.

Procedure	TVT	TOT	Anterior vaginal mesh	Sacrocolpopexy	Total	IUGA/ICS-classification
Number of patients (gender)	7 (female)	4 (male)	1 (female)	8 (female)	20	
Complications, number (%)						
Clavien-Dindo Grade I						
Prolonged pain	0	1 (25%)	0	1 (12.5%)	2 (10%)	6Be/S4
Hematoma	1 (14%)	1 (25%)	0	0	2 (10%)	7A/S3/S4
Urge *de novo *	3 (43%)	0	0	0	3 (15%)	4B/site?
Obstruction (prolonged cath.)	1 (14%)	0	1 (100%)	0	2 (10%)	4B/site?
Grade II						
UTI	2 (28%)	0	0	2 (25%)	4 (25%)	4B/site?
Grade III						
Obstruction (reoperation)	2 (28%)	0	0	0	2 (10%)	4B/S1
Bladder/bowel injury	0	0	0	0	0	4A/S3, 5A/B/S5
Fistula	0	0	0	0	0	4/5B/S1 or S2
Mesh exposure	0	0	0	0	0	2B or 3B/S1 or S2
QoL improved	6 (86%)	2 (50%)	1 (100%)	7 (87.5%)	16 (80%)	
